# End-Stage Renal Disease among Patients Undergoing Haemodialysis at a Tertiary Care Centre: A Descriptive Cross-sectional Study

**DOI:** 10.31729/jnma.7258

**Published:** 2022-05-31

**Authors:** Ajay Rajbhandari, Ujwal Bhusal, Dhan Bahadur Shrestha, Jyoti Yadav, Sonam Singh, Chiranjibi Pant, Arun Sharma

**Affiliations:** 1 Department of Internal Medicine, Shree Birendra Hospital, Chhauni, Kathmandu, Nepal; 2 Nepalese Army Institute of Health Sciences, Syanobharyang, Kathmandu, Nepal; 3 Department of Internal Medicine, Mount Sinai Hospital, Chicago, Illinois, United States of America; 4 Department of Emergency Medicine, Bir Hospital, Kanti Path, Kathmandu Nepal; 5 Department of Nephrology, Shree Birendra Hospital, Chhauni, Kathmandu, Nepal

**Keywords:** *chronic kidney failure*, *chronic renal insufficiency*, *end stage renal disease*, *hemodialysis*, *Nepal*

## Abstract

**Introduction:**

Chronic kidney disease is defined as structural or functional damage of the kidney persisting for three or more months. Studies have shown hypertension and diabetes as the leading causes of chronic kidney disease. The aim of this study is to find out the prevalence of end-stage renal disease patients undergoing haemodialysis in a tertiary care hospital.

**Methods:**

This was a descriptive cross-sectional study conducted among 96 patients undergoing haemodialysis from February 13, 2021 to April 4, 2021 in the hemodialysis unit of a tertiary care centre after receiving ethical clearance from the Institutional Review Committee (Reference number: 354). Convenience sampling was done and all patients older than 18 years who were on maintenance haemodialysis on an outpatient basis were included in the study. Data were collected using a selfadministered questionnaire. Data were analysed using the Statistical Package for the Social Science version 22.0. Point estimate at 95% Confidence Interval was calculated along with frequency and percentages for binary data and mean with standard deviation for continuous data.

**Results:**

Among 96 patients undergoing haemodialysis, the prevalence of end-stage renal disease was 83 (86.45%) (79.60-93.30 at 95% Confidence Interval). The most common underlying condition was hypertensive nephropathy in 34 (40.96%) patients, followed by both hypertensive and diabetic nephropathy in 26 (31.33%) patients.

**Conclusions:**

The prevalence of end-stage renal disease in our study was higher when compared to similar studies conducted in similar settings. Early diagnosis and adequate treatment of hypertension and diabetes could be crucial to reducing the prevalence of the end-stage renal disease.

## INTRODUCTION

Chronic Kidney Disease (CKD) is defined as kidney damage or an estimated Glomerular Filtration Rate (eGFR) less than 60 ml/min/1.73 m^2^ persisting for 3 months or more, irrespective of the cause.^1^ Globally, CKD cases are rising due to the increased prevalence of contributing illnesses such as hypertension and diabetes with the global prevalence of CKD was 9.1% as of 2017.^2^

The prevalence of CKD in Nepal is 6.0% among the general population^3^ and as high as 27.6% among high-risk populations.^4^ Various institutional studies of Nepal have shown hypertension and diabetes as the major causes of CKD with a prevalence of 24.5% and 5.8% respectively.^5-8^ The more widespread efforts at prevention, early detection, evaluation, and management of CKD and antecedent conditions could prevent complications of decreased kidney function.

The aim of this study is to find out the prevalence of end-stage renal disease among patients undergoing haemodialysis in a tertiary care hospital.

## METHODS

This was a descriptive cross-sectional study conducted among 96 patients undergoing haemodialysis from February 13, 2021 to April 4, 2021 in the hemodialysis unit of the Department of Nephrology of Shree Birendra Hospital, Chhauni, Kathmandu after receiving ethical clearance from the Institutional Review Committee (Reference number: 354). All patients with CKD and aged 18 years or more, who were on maintenance hemodialysis on an outpatient basis were included in the study. In contrast, patients with carcinoma involving one or both kidneys and patients who underwent renal transplants were excluded.

Convenience sampling was done and the sample size was calculated using the formula:

n = (Z^2^ × p × q) / e^2^

  = (1.96^2^ × 0.50 × 0.50) / 0.1^2^

  = 96

Where,

n = required sample size
Z = 1.96 at 95% Confidence Interval (CI)
p = prevalence taken as 50% for maximum sample size calculation
q = 1-p
e = margin of error, 10%

Hence, a sample size of 96 was considered for the study. Data was collected through a self-administered questionnaire. Detailed clinical history and general and systemic examination findings of all patients were reviewed. Clinical signs and symptoms pertaining to various systems affected by CKD were questioned. The diagnosis of ESRD was made considering clinical signs and symptoms of the patient, renal ultrasound, blood tests and Estimated Glomerular Filtration Rate (eGFR) which was calculated using the Modification of Diet in Renal Disease ( MDRD) formula:^9^

eGFR (ml/min/1.73 m^2^) = 175 × (Serum Creatinine)-1.154 × (age in years) - 0.203 × 0.742 (if female) × 1.212 (if black)

In all the study participants, Complete Blood Count (CBC), serum electrolytes, Renal Function Tests (RFTs), serum calcium, serum phosphorus, serum uric acid, serum protein, serum albumin, serum iron, serum ferritin, Total Iron Binding Capacity (TIBC), Chest X-ray, 2D-Echocardiogram (ECHO) records were evaluated. To find the coexisting conditions with ESRD, patients' history and previous laboratory reports (e.g., renal biopsy) were considered. Funduscopic findings were considered as supportive evidence to label diabetic and hypertensive nephropathy where applicable. In addition, Electrocardiogram (ECG) and 2D-ECHO findings of left ventricular hypertrophy were considered supporting evidence for hypertensive nephropathy.

Data were analysed using the Statistical Package for the Social Science version 22.0. Point estimate at 95% Confidence Interval was calculated along with frequency and percentages for binary data and mean with standard deviation for continuous data.

## RESULTS

Among 96 patients undergoing haemodialysis, the prevalence of end-stage renal disease was 83 (86.45%) (79.60-93.30 at 95% Confidence Interval). The mean age of the ESRD patients was 54.17±16.41 years. Fifty- two (62.66%) of them were males, and 31 (37.34%) were females (Table 1).

**Table 1:** Demographic details of the patients with end-stage renal disease (n = 83).

Variables		n (%)
**Sex**	Females	31 (37.34)
	Males	52 (62.66)
**Religion**	Buddhist	1 (1.20)
	Christian	2 (2.40)
	Hindu	80 (96.40)
**Marital status**	Married	82 (98.80)
	Unmarried	1 (1.20)
**Family type**	Joint	71 (85.54)
	Nuclear	12 (14.46)

The most common systemic manifestation complained by the patients was oliguria seen in 36 (43.38%) patients followed by muscle weakness in 35 (42.17%) patients, pedal edema in 33 (39.76%) patients, and breathlessness in 30 (36.15%) patients (Table 2).

**Table 2:** Clinical signs and symptoms of the patients with end-stage renal disease (n = 83).

Pedal edema	33 (39.76)
Breathlessness	30 (36.15)
Anorexia	19 (22.90)
Vomiting	24 (28.92)
Pleural effusion	3 (3.62)
Pulmonary edema	4 (4.82)
Bone pain	19 (22.90)
Muscle weakness	35 (42.17)
Oliguria	36 (43.38)
Flank pain	12 (14.46)
Altered sensorium	1 (1.20)
Convulsions	8 (9.64)
Hypertension	77 (92.78)
Pericardial effusion	1 (1.20)
Metabolic acidosis	3 (3.62)

**Figure 1:**
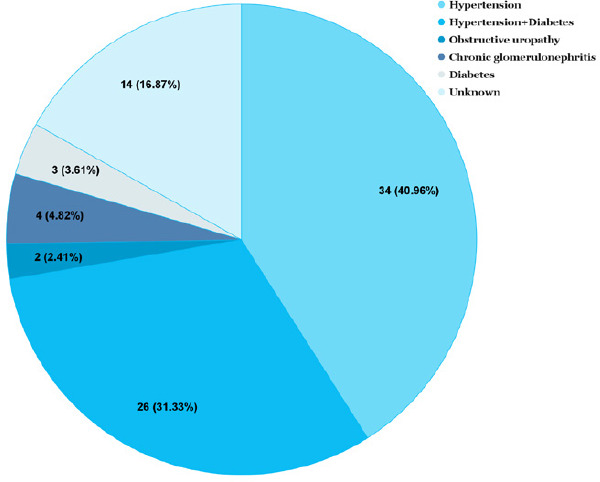
Comorbidities observed in patients with end-stage renal disease (n= 83).

The most commonly observed comorbidity among the patients was hypertension in 34 (40.96%), followed by hypertension with diabetes in 26 (31.33%) (Figure 1).

The mean duration of dialysis was 24.5±30.07 months. The average Urea Reduction Ratio (URR) was 59.07±21.48%. The mean haemoglobin level and Erythrocyte Sedimentation Rate (ESR) were 8.54±1.88 g/dl and 27.87±12.68 mm/hr. Similarly, the mean ferritin was 1090.96±705.66 mcg/l. Additionally, 83 (100%) patients were seronegative for Human Immunodeficiency Virus (HIV), Hepatitis B Surface Antigen (HBsAg) and Anti-hepatitis C Virus (Anti-HCV) antibodies. The laboratory parameters of the patients with end stage renal disease are tabulated below (Table 3).

**Table 3:** Laboratory parameters (n= 83).

	Before dialysis Mean±SD	After dialysis Mean±SD
Sodium (meq/l)	135.11±4.48	137.40±8.08
Potassium (meq/l)	5.35±.93.00	3.64±0.78
Calcium (mg/dl)	9.00±1.32	10.35±1.39
Phosphate (mg/dl)	6.45±2.51	3.45±1.60
Protein (g/dl)	6.96±.54.00	7.54±1.20
Albumin (g/dl)	3.65±.62.00	3.89±0.76
Urea (mg/dl)	179.06±50.90	74.21±47.90
Creatinine (mg/dl)	11.08±4.02	5.06±2.95

## DISCUSSION

Among 96 patients undergoing hemodialysis, 83 (86.45%) were on maintenance dialysis twice a week for ESRD whereas remaining 13 (13.55%) were emergency dialysis for various reasons. Our institution has been providing free hemodialysis service twice a week to ESRD patients under the Nepal government "Bipanna Nagarik Kosh". That may explain the high proportion (86.45%) of maintenance hemodialysis. Among the 83 ESRD patients, the average age was 54.16 years, the range being 20 to 88 years. This indicates that people of any age, from young adults to the elderly, suffer from chronic kidney disease requiring hemodialysis. This even reflects the increasing number of younger people in the working-age group suffering from kidney disease and the importance of early preventive measures to prevent progression to ESRD. This finding is consistent with several other studies from Nepal^5-7^ and India.^10,11^ This is different from the USA studies where the mean age of the patients was above 62 years.^12,13^

Unfortunately, only 30 (36.14%) of the total patients had URR more than 65% which is considered to be adequate for hemodialysis. The most common underlying condition of CKD in our study was hypertensive nephropathy 34 (40.96%), followed by both hypertensive as well as diabetic nephropathy 26 (31.33%), chronic glomerulonephritis 4 (4.82%), diabetic nephropathy 3 (3.61%), and obstructive uropathy 2 (2.41%). Those were the common risk factors of CKD as per a review of studies from the Nepalese population.^4^ Hypertension has been reported as a major cause of CKD in a study.^5^ Similar etiological proportions have been reported by various studies in different tertiary centres of Nepal.^6,7^ In contrast to ours, studies from India report diabetes as the most common underlying comorbidity followed by hypertension.^10,11^

The most common systemic manifestations in our study were oliguria followed by muscle weakness, pedal edema, and breathlessness. However, the most common manifestations in a study done in 2017 were breathlessness (80%), oliguria (76%), hypertension (74%), anorexia (38%), and muscle weakness (66%). Hypertension was found in 77 (92.77%) patients, similar to another study that reported hypertension in 84% of the patients.^14^ The most commonly involved system in our study was the excretory system, followed by the musculoskeletal system and fluid overload. Similar results of the most common system-wise manifestations being the excretory system and due to fluid overload accounting for 82% each, followed by the cardiovascular system (76%) and musculoskeletal system (74%) have been reported.^11^ Similarly, another study found excretory system manifestations in 85% of patients and gastrointestinal system involvement in 81.5% of the patients.^14^

Our study showed that oliguria is the most common symptom which could be due to the reduction of renal mass. Oliguric patients must follow salt and fluid restrictions to decrease the incidence of fluid overload presenting as pedal edema. Similarly, pedal edema could be associated with common cardiovascular risk factors such as older age and left ventricular hypertrophy. Muscle weakness and lack of endurance could be caused by uremic myopathy resulting in a sedentary lifestyle, which leads to progressive deconditioning and an increase in morbidity and mortality. Nonetheless, exercise could attempt to negate this. In our study, 30 (36.14%) patients had breathlessness that could be due to congestive heart failure or unrecognized chronic lung disease or dialyzer bio-incompatibility or anemia, or sodium and fluid overload.

The average hemoglobin level among the eighty-three patients was 8.54±1.87 g/dl with 65 (78.31%) having hemoglobin less than 10 g/dl. A study among 863 patients with ESRD for anemia found up to 90% of patients with hemoglobin less than 10 g/dl.^15^ Similarly, a longitudinal analysis involving >65,000 dialysis patients showed only approximately 38% had hemoglobin levels within the range of 11 to 12 g/dl.^16^

Hyperkalemia (serum potassium >5.5 meq/l) in pre-dialysis samples was prevalent in 38 (45.78%) patients in our study. Serum potassium level >6.0 mmol/l has been associated with higher rates of hospitalization and death than individuals with serum potassium <5 mmol/l.^17^ With the progression of CKD, potassium excretion progressively decreases. Intake of potassium-rich foods further burdens diseased kidneys with extra potassium leading to hyperkalemia. In addition to that, the state of metabolic acidosis in such patients promotes the extracellular shifting of potassium. Several drugs used by CKD patients such as Renin-Angiotensin-Aldosterone System (RAAS) inhibitors, p2-adrenergic receptors blockers, and cardiac glycosides also elevate serum potassium levels. Hyponatremia was prevalent among 42/83 (50.60%) of the patients, whereas hypernatremia was found in only one patient. Hyponatremia could be a consequence of fluid overload or a result of diuretic usage in these patients.

There were some limitations in this study. First of all, it was a study conducted in a single tertiary centre involving a small sample size which can affect the generalisability of the findings. Secondly, it is a simple descriptive study that cannot prove causality or association. Multicentre studies involving a large number of patients are necessary to better understand the distribution, etiology, and clinical manifestations of ESRD among the Nepalese population.

## CONCLUSIONS

The prevalence of end-stage renal disease in our study was higher when compared to similar studies conducted in similar settings. The majority of the cases had diabetes and hypertension. Therefore, early diagnosis and treatment with proper control of blood pressure and blood sugar level from the early stages of these diseases could slow and even prevent the progression of kidney damage. Furthermore, primary prevention with health education and specific protection from a young age could decrease the disease prevalence substantially.
